# Seeing Central African forests through their largest trees

**DOI:** 10.1038/srep13156

**Published:** 2015-08-17

**Authors:** J.-F. Bastin, N. Barbier, M. Réjou-Méchain, A. Fayolle, S. Gourlet-Fleury, D. Maniatis, T. de Haulleville, F. Baya, H. Beeckman, D. Beina, P. Couteron, G. Chuyong, G. Dauby, J.-L. Doucet, V. Droissart, M. Dufrêne, C. Ewango, J.F. Gillet, C.H. Gonmadje, T. Hart, T. Kavali, D. Kenfack, M. Libalah, Y. Malhi, J.-R. Makana, R. Pélissier, P. Ploton, A. Serckx, B. Sonké, T. Stevart, D.W. Thomas, C. De Cannière, J. Bogaert

**Affiliations:** 1 Landscape Ecology and Plant Production Systems Unit, Université libre de Bruxelles, CP264-2, B-1050 Bruxelles, Belgium; 2 BIOSE Department, Gembloux Agro-Bio Tech, Université de Liège, B-5030 Gembloux, Belgium; 3 Ecole Régionale post-universitaire d’Aménagement et de gestion Intégrés des Forêts et Territoires tropicaux, Kinshasa, DR Congo; 4 UMR AMAP, IRD, F-34000 Montpellier, France; 5 UPR BSEF, CIRAD, Campus International de Baillarguet, F-34398 Montpellier, France; 6 Environmental Change Institute, School of Geography and the Environment, University of Oxford, Oxford, UK; 7 Laboratory for Wood Biology and Xylarium, Royal Museum for Central Africa, Tervuren, Belgium; 8 Ministère des Eaux, Forêts, Chasse et Pêche, BP 3314 Bangui, Central African Republic; 9 Université de Bangui – Cerphameta, BP 1450 Bangui, Central African Republic; 10 Department of Plant and Animal Sciences, University of Buea, P.O. Box 63, Buea, Cameroon; 11 Laboratoire d’Evolution Biologique et Ecologie, Faculté des Sciences, Université libre de Bruxelles, CP160-12, Brussels, Belgium; 12 Centre de Formation et de Recherche en Conservation Forestière (CEFRECOF), Wildlife Conservation Society, Kinshasa, DR Congo; 13 National Herbarium, P.O BOX 1601, Yaoundé, Cameroon; 14 CIFOR, Central African Regional Office, P.O. Box 2008 Messa, Yaoundé, Cameroon; 15 Lukuru Wildlife Research Foundation, Kinshasa, Gombe, DR Congo; 16 Wildlife Conservation Society - DRC Program, Kinshasa, DR Congo; 17 CTFS-ForestGEO, Department of Botany, MRC 166, Smithsonian Institution, P.O. Box 37012, Washington, DC 20013-7012, USA; 18 Behavioural Biology Unit, University of Liege, Liege, Belgium; 19 Conservation Biology Unit, Royal Belgian Institute of Natural Sciences, Brussels, Belgium; 20 Department of Primatology, Max Planck Institute for Evolutionary Anthropology, Leipzig, Germany; 21 Plant Systematic and Ecology Laboratory, Department of Biology, University of Yaounde 1, Yaounde, Cameroon; 22 Missouri Botanical Garden, Africa and Madagascar Department, St Louis, MO, USA; 23 Department of Botany and Plant Pathology, Oregon State University, Corvallis, OR 97331, USA

## Abstract

Large tropical trees and a few dominant species were recently identified as the main structuring elements of tropical forests. However, such result did not translate yet into quantitative approaches which are essential to understand, predict and monitor forest functions and composition over large, often poorly accessible territories. Here we show that the above-ground biomass (AGB) of the whole forest can be predicted from a few large trees and that the relationship is proved strikingly stable in 175 1-ha plots investigated across 8 sites spanning Central Africa. We designed a generic model predicting AGB with an error of 14% when based on only 5% of the stems, which points to universality in forest structural properties. For the first time in Africa, we identified some dominant species that disproportionally contribute to forest AGB with 1.5% of recorded species accounting for over 50% of the stock of AGB. Consequently, focusing on large trees and dominant species provides precise information on the whole forest stand. This offers new perspectives for understanding the functioning of tropical forests and opens new doors for the development of innovative monitoring strategies.

Large trees play an important role in forest structure, functioning and diversity^1^. They provide nesting sites and shelter for up to 30% of all vertebrate species^2^, produce food^1^ and support a wide diversity of epiphytes and lianas. Towering above the canopy, large trees both enhance and regulate forest regeneration and species coexistence by attracting dispersers, pollinators, herbivores and pathogens^3^,^4^. They influence understory species composition by preempting light and impacting local microclimates^5^ and their dead material can persist for decades which provides key habitat for ground fauna. And recently because large trees play a major role in the global carbon cycle^1^, they have become a focus of forest carbon research^6^,^7^,^8^,^9^.

In tropical forests, large trees concentrate a large fraction of forest carbon stocks within their above-ground biomass (AGB)^10^, and they accumulate carbon faster than smaller trees^8^. Therefore, the AGB of the largest tropical trees may be a good indicator of AGB dynamics as a whole. For instance, the density of trees with a diameter at breast height (DBH) greater than or equal to 70 cm explained almost 70% of the variation in AGB among 120 pantropical sites^6^. However, large trees represent a small fraction of all tree individuals, so that they are easily over or under-represented by small sampling plots, potentially leading to high sampling errors in AGB estimation^11^. The metabolic scaling theory^12^,^13^ predicts that the inverse relationship between tree size and abundance does not vary within natural forests, i.e. here considered as a forest whose dynamics is only driven by ecological processes. Therefore, the structure of the entire forest can be approximated from the abundance of trees in a given size class. Both theoretical and empirical approaches^13^,^14^,^15^ have shown that non-competition induced mortality (e.g., mortality due to drought, fire or wind blowdown events^1^,^16^) could lead to a systematic overestimation of the density of large trees when using the metabolic scaling theory. Yet, because these deviations do appear to be systematic^13^, the density of large trees should still convey information about the structure of the understory tree community.

Recent studies conducted on the entire Amazonian basin showed that a small number of species contribute disproportionately to the global stem density and biomass, as they estimated that only 1.4% of tree species account for the half of the regional stem abundance^17^ and only 0.91% of tree species account for the half of the regional AGB^18^. These species were considered respectively as ‘stem hyperdominant’ and ‘biomass hyperdominant’. Identifying these key species is important to better understand the structure and the functioning of tropical forests^19^, and to develop effective monitoring and conservation strategies. This is of particular importance in Central Africa, the second largest area of continuous rainforest in the world (after the Amazonian basin), and reported as the less studied^20^.

We used a dataset of 175 1-ha field plots established in natural stands of moist tropical forests and scattered across 8 sites from western Cameroon to eastern Democratic Republic of the Congo (Fig. 1, and see Supplementary Table S1 online), to answer the following questions: Do the largest trees mirror the AGB and diversity of the entire forest? How does Central African biomass hyperdominance compare with that in the Amazon? These questions are critical to the understanding of tropical forest ecology and the development of cost-efficient forest monitoring programs.

**The forest viewed through its largest trees**

We first investigated the accumulation of AGB and species richness of trees ranked by decreasing size. For each site, the mean proportion of the total AGB per plot (hereafter AGB_TOT_) increased rapidly with the cumulative AGB of the largest trees (Fig. 2A), reaching an average of 50% (s.d. 10%) for the 20 largest trees (approximately 5% of the stems) and 82% (s.d. 8%) for the 100 largest trees (approximately 25% of the stems). The concentration of AGB in a limited number of trees has previously been observed^6^,^10^, but both the steepness of the slope of AGB accumulation and the remarkable constancy of the observed trends across study sites was unexpected. Using the AGB of the largest trees to predict AGB_TOT_, we showed that the coefficient of determination (R^2^) increased asymptotically with the cumulative number of trees being considered (Fig. 2B). The AGB of the individual largest tree in each plot (AGB_top1_) explained 48% of the variance in AGB_TOT_ across all plots (relative residual standard error (RSEr) of approximately 28%). This is in agreement with Stegen *et al.*^21^ results, where they consistently showed from 275 0.1-ha plots spanning North and South America that the AGB_top1_ has an important influence on the total stand biomass. AGB_top10_ explained 77% of the variance (RSEr of approximately 19%), and 87% was attained with AGB_top20_ (RSEr of approximately 14%). In other words, only measuring approximately 5% of the stems in a 1-ha plot allows for an estimate of the entire AGB with close to 90% precision (Fig. 2C, and see Supplementary Table S2 online). A leave-one-out cross-validation of the regional model based on AGB_top20_, i.e., calibrating the regional models without one site used to validate the model, gave an average Pearson’s correlation of 0.94 and a bias of only 15%. This result shows that the biomass of the entire forest can be extrapolated from the biomass of the largest trees with relatively small levels of uncertainty. Consequently, we developed a generic model specific to Central Africa to predict AGB_TOT_ (in kg/ha) from the AGB of the largest trees (Equation 1 and See Supplementary Table S3 online). For ease of use, we also developed two equations to estimate the coefficients of the generic model from the number of largest trees considered (N), with a range of validity between the 5 and the 100 largest trees (Equation 2 and Equation 3, and see Supplementary Fig. S1 online). Note that the model parameters were robust to the allometric biomass equation used to estimate AGB (see Supplementary Fig. S2 online).


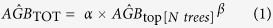



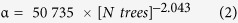



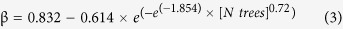


The total species richness, species_TOT,_ was also rather well represented by the largest trees, but the relationship was weaker and less stable than for AGB_TOT_. The mean proportion of species_TOT_ represented by the largest trees rose much less rapidly than for AGB. Most of the sites showed an asymptotic increase in species richness with the number of trees considered (Fig. 2D). The Ituri site showed a singular S-shaped curve caused by the dominance of *Gilbertiodendron dewevrei*, a species known to form nearly monodominant stands^22^. For all sites, the 20 largest trees accounted for approximately 20% of species_TOT_ (s.d. 5%), and the 100 largest trees accounted for approximately 45% (s.d. 10%). The accuracy of the species_TOT_ prediction from the largest trees also increased asymptotically with the number of trees considered (Fig. 2E) with the exception of the Mbaïki site, which showed particularly little variation in species_TOT_ between plots (from 120 to 160). At the site level, the 20 largest trees explained from 10 to 80% of species_TOT_ (48% when including all of the sites). In contrast to AGB, the parameters of the species_TOT_ prediction models were highly variable across sites, as illustrated by the heterogeneity of the slopes and intercepts between the sites using the species richness of the 20 largest trees as the predictor variable (Fig. 2F).

**AGB hyperdominance**

Finally, we sought to measure the ‘biomass hyperdominance’ in Central Africa, i.e. the disproportionate contribution to biomass of a small number of species. Using an approach similar to that of Ter Steege *et al.*^17^, but applied on species AGB instead of the number of stems, we studied both regional ‘biomass hyperdominance’ and local ‘biomass dominance’ patterns. Here, we found that only 18 species (out of 1194 recorded; i.e. 1.5%) accounted for 50% of the total AGB of our dataset (Table 1, and see Supplementary Table S4 and Table S5 online), and that, at the site level, only 4.4% (s.d. 1.8%) of species accounted for 50% of the AGB on average. The difference of proportion is explained by the strong overlap between ‘local dominant’ and ‘regional hyperdominant’ species. Two sites present however a large proportion of ‘local dominant’ species not found elsewhere: Mbaïki and Ngovayang (Fig. 3), which both contain typical species of the southern part of the Central African Republic (e.g. *Manilkara spp.*)^23^ or of mountain forests (e.g. *Strombosia scheffleri*)^24^ respectively.

Our findings are consistent with the results recently obtained in the highly diverse Amazonian basin, where a strong species hyperdominance has been found both in term of stem density^3^ and biomass^23^ and where a scaling-up from local dominance to the regional hyperdominance was also observed^17^. It should be however acknowledged that our sampling design, i.e. 175 plots grouped in 8 sites, may have overestimated the dominance of species due to spatial autocorrelation in their abundances, even if dominant species tend to be less spatially aggregated than rare species^25^. The regional ‘hyperdominance’ is most likely to occur in all tropical regions (e.g. in south-eastern Asia), and may serve as a basis to better understand important ecological and functional differences between the main tropical forest regions of the world.

In Africa, several of the “biomass hyperdominant” species we identified are frequently (*Lophira alata, Erythrophleum suaveolens, Staudtia kamerunensis*) or ‘occasionally’ (*G. dewevrei, Klainedoxa gabonensis, Desbordesia glaucescens, Dialium pachyphyllum*) logged^26^. Logging activities in those regions may thus lead to an important reduction of the current carbon stocks in Central Africa where approximately 26% of the forested area is currently managed under logging concessions^26^. Interestingly, 7 of the 18 biomass hyperdominant species in the present study are typically middle-sized trees that rarely reach large diameters (e.g., 70 cm): *Coula edulis, Oubanguia alata, Plagiostyles africana, Polyalthia suaveolens, Strombosia pustulata, Scorodophleus zenkeri,* and *S. kamerunensis*. While Fauset *et al.*^18^ showed that Amazonian “biomass hyperdominant” species tend to have a large maximum size, our result show that very common and abundant middle-sized tree species also play a key role in the carbon budget.

## Conclusions

In this study, we showed that the largest trees accurately and consistently represent the AGB of the entire forest across a range of contrasting sites covering Central Africa. Despite consistent deviations from the metabolic scaling theory predictions reported in the literature^13^,^14^,^15^, largest trees can still predict the biomass of forest stands over vast areas and, as suggested by Stegen *et al.*^21^, across contrasted climatic and environmental conditions. This offers a unique opportunity to greatly improve the cost-effectiveness of field programs needed to implement international climate change mitigation policies in tropical forests, and to minimise uncertainties related to remote-sensing products aiming to predict the biomass of the forest^27^,^28^ by focusing on objects directly observable from space, i.e. the largest trees (see Supplementary Note online). Scientific permanent plots, monitoring trees regardless of their size are however still required to understand forest dynamics, diversity and functioning at high spatial resolution.

Finally, we showed that the forests investigated in Central Africa present similar patterns of ‘biomass hyperdominance’ to those found in the Amazon, with 1.5% of the recorded species accounting for 50% of the regional AGB. The loss of these ‘hyperdominant’ species, for instance through forest logging activity, may prove to be a important issue in the near future for both the ecosystems functions they support and forest carbon storage^19^. Their identification should therefore constitute a priority as they offer the opportunity to understand key ecological and functional differences between the tropical regions and to develop appropriate conservation strategies.

## Methods

### Dataset

A combination of recently inventoried existing permanent designs and non-permanent large plots (1-ha) was used: Ituri-Lenda (20 ha; Democratic Republic of Congo), Korup (50 ha; Cameroon), Korup2 (2 ha; Cameroon), Lomie Kongo (3 ha; Cameroon), Mabounie (12 ha; Gabon), Malebo (31 ha; Democratic Republic of Congo), Mbaïki (12 ha; Central African Republic), Mindourou (10 ha; Cameroon), Ngovayang massif (15 ha; Cameroon) and Yangambi (20 ha; Democratic Republic of Congo). Plots larger than 1-ha were subdivided into 1-ha plots. In total, our analyses relied on 175 1-ha plots.

### Field measurements

Diameter at breast height (DBH, measured at 130 cm or 50 cm above any buttresses) has been measured for all trees with a DBH greater than or equal to 10 cm. Measured trees were identified up to the species-level in the field, and samples were deposited in different herbarium collections (see Supplementary Table S1 online). Tree height was directly integrated in the estimations of AGB when measured, i.e. using multiple measurements with an hypsometer to estimate the height between the top of the crown and the bottom of the tree (Malebo). When only a subset was measured, we used site-specific height-diameter allometries to estimate the height of all the trees (Korup, Mabounie, SE Cameroon, Yangambi).

### AGB estimates

We used the allometric equations developed by Chave *et al.*^29^ for moist tropical forests (with and without tree height in the set of predictors depending on the availability of height data and the site-specific height-diameter allometry).

### Wood specific gravity

We used the Dryad repository^30^ to obtain the wood specific gravity values for each species (using genus or family averages if species-level information was not available).

### Cumulative trees ranked by decreasing size

Trees were ranked in each plot by decreasing size according to their AGB. The prediction of total AGB and species richness from the largest trees was done using iterative predictions from an incremental, cumulative method from the largest to the smallest tree in each plot.

### AGB prediction from the largest trees

To predict AGB_TOT_ from the AGB of the largest trees, we computed power regression models with no intercept:


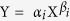


The value of the power model coefficient (*α*) is predicted from the number (*i*) of the largest trees considered using a power regression model with no intercept:


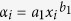


The value of the power model exponent, β, is predicted from the number (*i*) of the largest trees considered using a Weibull model as follows:


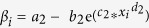


### AGB hyperdominance

We considered species as being ‘biomass hyperdominant’, the first species that cumulate 50% of the total AGB at a regional scale, when ranked by decreasing contribution to the total AGB. To avoid any bias due to different number of plots used in the different sites, the contribution of each species was standardized by site. Therefore, each site contributes equally to the regional AGB. ‘Local dominance’ was quantified similarly at the site level.

Statistical analyses were performed using the open-source software R (http://cran.r-project.org).

## Additional Information

**How to cite this article**: Bastin, J.-F. *et al.* Seeing Central African forests through their largest trees. *Sci. Rep.* **5**, 13156; doi: 10.1038/srep13156 (2015).

## Supplementary Material

Supplementary Information

## Figures and Tables

**Figure 1:**
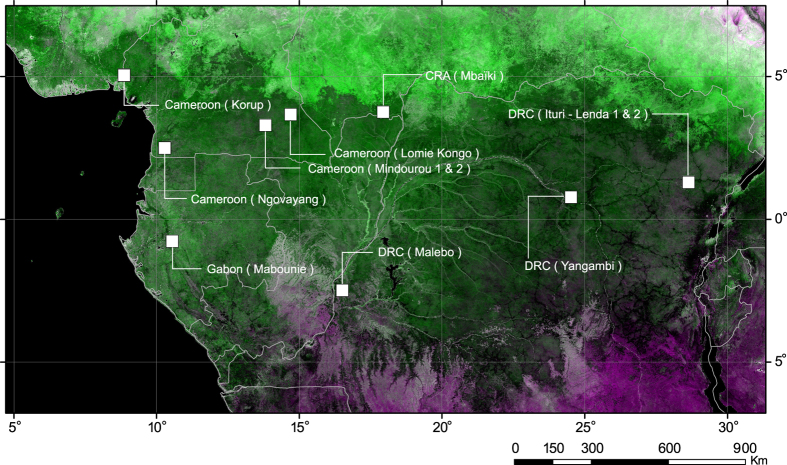
Site locations. Spatial distribution of the study sites superimposed in white on a false color of Enhanced Vegetation Index (EVI) composite map centred on Central Africa. The map was produced from a yearly synthesis from twelve MODIS-EVI 250 m data (MOD13Q1 c5). The 8th, 1st and 8th, 16-day periods are projected in red, green and blue color channels. Copyright Dr. Valery Gond.

**Figure 2:**
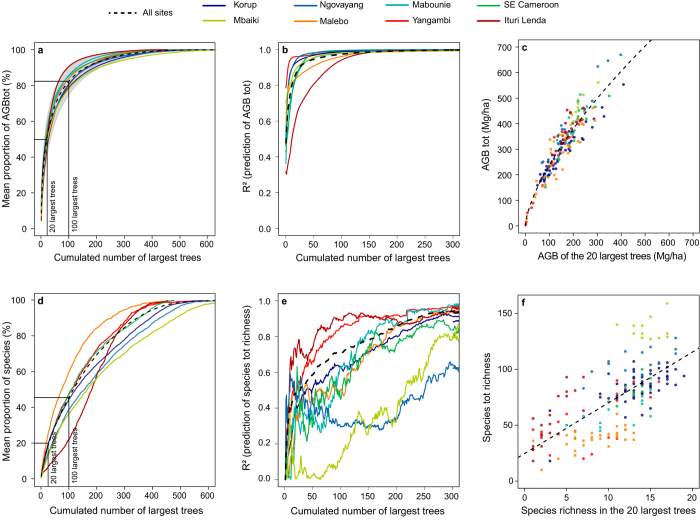
Proportion and prediction of the entire above-ground biomass (AGB_TOT_) and species richness (species_TOT_) from the largest trees. Results are displayed for the entire dataset (black dotted-line) superimposed on each study site (coloured lines). Larger trees store most of the AGB_TOT_ (**a**) and predict most of the AGB_TOT_ variance among plots (**b**) up to an R^2^ of 0.87 for the 20 largest trees (**c**). Species richness is generally high among the largest trees but depends on forest type (**d**) as shown by the S-shaped curve of the Ituri site, which corresponds to the monodominant *Gilbertiodendron dewevrei* forests. Species richness of the largest trees often predicts a non-negligible share of total species richness (**e**) but is strongly dependent on site location (**f**).

**Figure 3:**
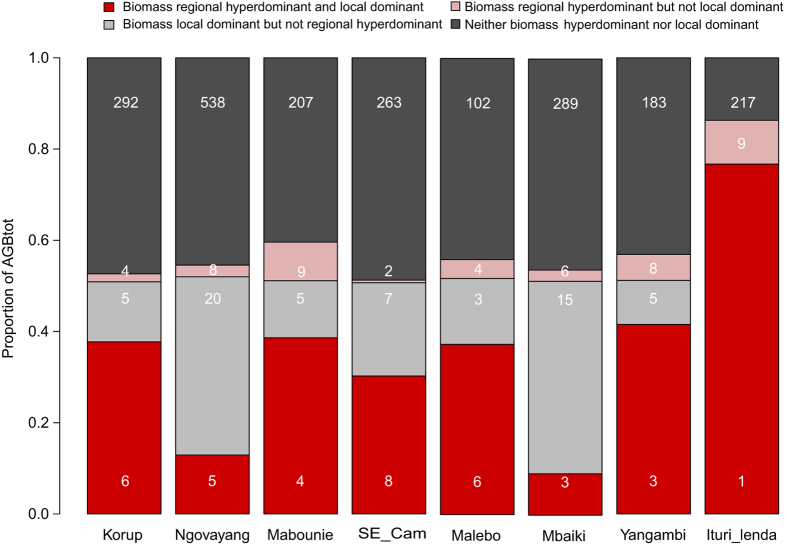
Proportion of AGB hyperdominant species by location. The sums in the red (local and regional AGB Hyperdominant species) and light grey (local but not regional AGB Hyperdominant species) barplots correspond to the sum of the local AGB dominant species (≥50% of AGB_TOT_). White integers correspond to the number of species in each fraction.

**Table 1:** Biomass Hyperdominant species (cumulating 50% of total AGB) of the 8 sites investigated (Kor = Korup; Ngov = Ngovayang; Mab = Mabounie; SE_Cam = South-East Cameroon; Mba = Mbaïki; Mal = Malebo; Yan = Yangambi; Itu = Ituri-lenda).

**Species**	**Family**	**AGB (t/ha) regional**	**% AGB (t/ha) regional**	**% cumulated AGB (t/ha) regional**	**Nb of sites**	**Local Site BHD**
Gilbertiodendron dewevrei**	*Fabaceae*	46.64	20.02	20.02	3	Mal, Yan, It
Klainedoxa gabonensis**	*Irvingiaceae*	8.38	3.60	23.62	7	Kor, Mab, SE_Cam, Mal, Mbaïki, Yan, Itu
*Coula edulis*	*Olacaceae*	6.39	2.74	26.36	2	Kor, Mab, Ngov
Desbordesia glaucescens**	*Irvingiaceae*	6.33	2.72	29.08	3	Kor, Mab, SE_Cam
Dialium pachyphyllum**	*Fabaceae*	5.40	2.32	31.40	6	Kor, Ngov, Mab, SE_Cam, Mal, Yan
*Lecomtedoxa klaineana*	*Sapotaceae*	4.62	1.98	33.38	1	Kor
*Oubanguia alata*	*Lecythidaceae*	4.23	1.81	35.19	1	Kor
*Strombosia pustulata*	*Olacaceae*	4.22	1.81	37.00	6	Kor, Mab, SE_Cam, Mal, Yan, It
Lophira alata*	*Ochnaceae*	4.18	1.79	38.80	2	Kor, Mab
*Petersianthus macrocarpus*	*Lecythidaceae*	4.14	1.78	40.58	6	Mab, SE_Cam, Mal, Yan, Itu, Mbaïki
*Polyalthia cf.suaveolens*	*Annonaceae*	4.1	1.76	42.34	7	Ngov, Mab, SE_Cam,Mba, Mal, Yan, Itu
*Scorodophloeus zenkeri*	*Fabaceae*	4.07	1.74	44.08	3	Mab,Mal,Yan
*Julbernardia seretii*	*Fabaceae*	3.33	1.43	45.51	3	Yan, Itu
*Alstonia boonei*	*Apocynaceae*	2.88	1.24	46.75	5	Kor, Mab, SE_Cam, Yan, Itu
*Pentaclethra macrophylla*	*Fabaceae*	2.81	1.21	47.95	5	Mab, SE_Cam, Mal, Yan, Itu
Erythrophleum suaveolens*	*Fabaceae*	2.40	1.02	48.97	4	SE_Cam, Mal, Yan, Itu
Staudtia kamerunensis*	*Myristicaceae*	2.30	0.99	49.96	8	all
*Plagiostyles africana*	*Euphorbiaceae*	2.30	0.99	50.95	3	Mab, Mal, Ngov

^*^timber species.

^**^Occasional timber species.
