# Supplementary material for: Seeing Central African forests through their largest trees
Source: Sci Rep. 2015 Aug 17;5:13156. doi: 10.1038/srep13156 (PMC4538397; doi:10.1038/srep13156)
Supplement: Supplementary Information [file srep13156-s1.pdf]

SUPPLEMENTARY INFORMATION

Title

Seeing Central African forests through their largest trees

Authors

Bastin J.-F.*, Barbier N., Réjou-Méchain M., Fayolle A., Gourlet-Fleury S., Maniatis D., de Haulleville T., Baya F., Beeckman H., Beina D., Couteron P., Chuyong G., Dauby G., Doucet J.-L, Droissart V., Dufrêne M., Ewango C., Gillet J.F., Gonmadje C.H., Hart T., Kavali T., Kenfack D., Libalah M., Malhi Y., Makana J.-R., Pélissier R , Ploton P., Serckx A., Sonké B., Stevart T., Thomas D.W., De Cannière C. & Bogaert J.

*corresponding author

1) Supplementary Material

Supplementary Table S1| Metadata of investigated sites. Information is provided on the location, the model used to estimate forest AGB, the number of plots realized and the team responsible of the plot. CTFS refers to the Center for Tropical Forest Science, IRD refers to the Institute for Research and Development and CIRAD to the Center for Agricultural Research for Development.

Site	Country	X coord	Y coord	AGB estimation model	Tree height included in AGB estimation model	Area (ha)	Team
Korup	Cameroon	5°3'N	8°52'E	Chave (2005) - wet	yes, through local DBH - H allometry	50 + 2	CTFS + IRD
Ngovayang	Cameroon	3°15'N	10°36'E	Chave (2005) - moist	yes, through local DBH - H allometry	15	Gonmadje C.
Mabounie	Gabon	0°46'S	10°33'E	Chave (2005) - moist	yes, through local DBH - H allometry	12	IRD
SE Cameroon	Cameroon	3°31'N	14°17'E	Chave (2005) - moist	yes, through local DBH - H allometry	13	IRD
Malebo	Dem. Rep. of the Congo	2°29'S	16°34'E	Chave (2005) - moist	yes	32	Bastin J.-F., C De Cannière, J. Bogaert
Mbaïki	Central Rep. of Africa	3°47'N	17°57'E	Chave (2005) - moist	no	12	CIRAD
Yangambi	Dem. Rep. of the Congo	0°46'N	24°30'E	Chave (2005) - moist	yes, through local DBH - H allometry	19	De Haulleville T., H. Beeckman, J. Bogaert
Ituri-lenda	Dem. Rep. of the Congo	1°18'N	28°37'E	Chave (2005) - moist	no	20	CTFS

2) Supplementary Results

Supplementary Table S2| Total Above-ground biomass (AGBtot; kg) per plot and the correspondent Above-ground biomass of the 20 largest trees (AGBtop20; kg). The data is presented for the 175 plots and the corresponding 8 sites investigated across Central Africa.

plot	Site	AGBtop20 (kg)	AGBtot (kg)	plot	Site	AGBtop20 (kg)	AGBtot (kg)
1	Malebo	184609	313723	31	Malebo	120256	198558
2	Malebo	93064	226985	32	Malebo	116033	243063
3	Malebo	187009	307082	33	Yangambi	4082	15252
4	Malebo	149801	308207	34	Yangambi	2172	5143
5	Malebo	168860	345363	35	Yangambi	32475	89517
6	Malebo	179853	311983	36	Yangambi	8432	50812
7	Malebo	229306	327250	37	Yangambi	25687	70991
8	Malebo	237017	400226	38	Yangambi	71703	158514
9	Malebo	156976	287101	39	Yangambi	120709	346966
10	Malebo	172754	357116	40	Yangambi	164349	365271
11	Malebo	242187	395948	41	Yangambi	150997	323300
12	Malebo	289766	412391	42	Yangambi	155596	353021
13	Malebo	227156	409920	43	Yangambi	111167	251988
14	Malebo	282017	461420	44	Yangambi	166969	334888
15	Malebo	103081	337543	45	Yangambi	145685	295244
16	Malebo	107210	285890	46	Yangambi	121129	277652
17	Malebo	106747	235144	47	Yangambi	223140	397060
18	Malebo	77156	188472	48	Yangambi	153252	368150
19	Malebo	155479	220395	49	Yangambi	172568	363380
20	Malebo	46419	175530	50	Yangambi	164785	321151
21	Malebo	5849	27449	51	Yangambi	198022	349431
22	Malebo	44824	124960	55	Mabounie	132206	335403
23	Malebo	71391	154747	56	Mabounie	178204	318711
24	Malebo	106026	299855	57	Mabounie	192204	361150
25	Malebo	83657	161841	58	Mabounie	256681	466514
26	Malebo	129082	183115	59	Mabounie	246026	446780
27	Malebo	179434	284484	60	Mabounie	169614	326227
28	Malebo	95491	189982	63	Mabounie	139710	320127
29	Malebo	40269	112572	64	Mabounie	182456	423981
30	Malebo	220019	331153	65	Mabounie	199130	361210

plot	Site	AGBtop20 (kg)	AGBtot (kg)	plot	Site	AGBtop20 (kg)	AGBtot (kg)
66	Mabounie	229776	513746	91	Korup	145431	283486
67	Mabounie	80878	192929	92	Korup	148027	321684
68	Mabounie	106574	206007	93	Korup	95156	250584
52	SE Cameroon	219780	427616	94	Korup	99616	199966
53	SE Cameroon	203285	333017	95	Korup	85898	218300
54	SE Cameroon	204827	372901	96	Korup	88850	232867
69	SE Cameroon	223221	451822	97	Korup	93736	194662
70	SE Cameroon	139029	356506	98	Korup	182332	369964
71	SE Cameroon	176385	357072	99	Korup	134970	258876
72	SE Cameroon	138474	264314	100	Korup	77978	203411
73	SE Cameroon	349983	508525	101	Korup	100310	233696
74	SE Cameroon	254309	418352	102	Korup	80905	185975
75	SE Cameroon	140866	258450	103	Korup	181756	300910
76	SE Cameroon	291887	465946	104	Korup	79430	180403
77	SE Cameroon	83631	151650	105	Korup	64570	177076
78	SE Cameroon	236920	458856	106	Korup	125158	244007
61	Korup	167621	296280	107	Korup	328573	428573
62	Korup	286695	418500	108	Korup	134151	251478
79	Korup	147998	298961	109	Korup	101316	216025
80	Korup	120258	262316	110	Korup	50578	158943
81	Korup	253056	407875	111	Korup	63191	176319
82	Korup	198932	367656	112	Korup	95377	225863
83	Korup	157325	324011	113	Korup	72414	185306
84	Korup	116637	236467	114	Korup	320456	463764
85	Korup	111609	242049	115	Korup	96959	198560
86	Korup	157360	337674	116	Korup	127778	223546
87	Korup	112206	277356	117	Korup	184321	324539
88	Korup	147026	330201	118	Korup	409535	553223
89	Korup	204571	346517	119	Korup	81674	204011
90	Korup	191738	332261	120	Korup	68774	186225

plot	Site	AGBtop20 (kg)	AGBtot (kg)	plot	Site	AGBtop 20 (kg)	AGBtot (kg)
121	Korup	79076	174134	150	Ngovayang	302389	623909
122	Korup	186982	293835	151	Ngovayang	136570	322520
123	Korup	236873	342461	152	Ngovayang	154006	378008
124	Korup	62373	201695	153	Ngovayang	345084	646632
125	Korup	90011	217577	154	Ngovayang	74308	250880
126	Korup	116764	223099	155	Ngovayang	163962	377235
127	Korup	279461	385912	156	Ngovayang	211711	407453
128	Korup	241652	337415	157	Ngovayang	136982	293573
129	Ituri_lenda	127661	292882	158	Ngovayang	279118	488867
130	Ituri_lenda	174830	342059	159	Ngovayang	398671	669451
131	Ituri_lenda	237690	415813	160	Ngovayang	150086	392927
132	Ituri_lenda	192782	380787	161	Ngovayang	69447	247110
133	Ituri_lenda	242166	461288	162	Ngovayang	151647	306240
134	Ituri_lenda	228822	410310	163	Ngovayang	219649	487972
135	Ituri_lenda	188446	441481	164	Mbaiki	188780	396146
136	Ituri_lenda	247736	456153	165	Mbaiki	242464	512487
137	Ituri_lenda	138664	275664	166	Mbaiki	219630	463143
138	Ituri_lenda	154196	323096	167	Mbaiki	304096	561205
139	Ituri_lenda	218999	417679	168	Mbaiki	220120	478757
140	Ituri_lenda	215205	405684	169	Mbaiki	221102	492340
141	Ituri_lenda	168316	366875	170	Mbaiki	221288	417280
142	Ituri_lenda	188394	444413	171	Mbaiki	195091	434300
143	Ituri_lenda	135339	350717	172	Mbaiki	149640	355981
144	Ituri_lenda	174493	453197	173	Mbaiki	201649	408980
145	Ituri_lenda	167587	402203	174	Mbaiki	211217	446070
146	Ituri_lenda	183823	346487	175	Mbaiki	118558	329574
147	Ituri_lenda	198022	401618				
148	Ituri_lenda	152083	324434				
149	Ngovayang	96337	259516				

Supplementary Table S3| Above-ground biomass (AGBtot; in kg) prediction model parameters (coefficient α and exponent β) along the cumulated number of largest trees.

The value of each coefficient is calculated based on the entire dataset of 175 1-ha plot.

Number of largest trees	α	β	RSE	rRSE	R^2	Number of largest trees	α	β	RSE	rRSE	R^2
1	18587.7	0.283	91027	28.4	0.48	26	58.0	0.714	39358	12.3	0.90
2	7693.6	0.352	84977	26.6	0.55	27	53.9	0.719	38662	12.1	0.91
3	4367.6	0.394	80381	25.1	0.59	28	50.4	0.724	38010	11.9	0.91
4	2780.5	0.428	76191	23.8	0.63	29	47.2	0.728	37389	11.7	0.91
5	1866.1	0.459	72762	22.7	0.67	30	44.5	0.733	36812	11.5	0.91
6	1326.6	0.484	69712	21.8	0.69	31	42.0	0.736	36268	11.3	0.92
7	979.5	0.507	66977	20.9	0.72	32	39.8	0.740	35756	11.2	0.92
8	741.1	0.528	64386	20.1	0.74	33	37.8	0.744	35269	11.0	0.92
9	579.0	0.546	62023	19.4	0.76	34	35.9	0.747	34794	10.9	0.92
10	461.5	0.563	59840	18.7	0.77	35	34.1	0.751	34358	10.7	0.93
11	376.8	0.578	57845	18.1	0.79	36	32.5	0.754	33942	10.6	0.93
12	313.6	0.592	55995	17.5	0.80	37	31.0	0.757	33547	10.5	0.93
13	264.9	0.604	54283	17.0	0.81	38	29.7	0.760	33173	10.4	0.93
14	224.6	0.616	52632	16.4	0.83	39	28.5	0.763	32807	10.3	0.93
15	192.9	0.627	51130	16.0	0.84	40	27.3	0.766	32451	10.1	0.93
16	168.2	0.637	49760	15.6	0.84	41	26.3	0.769	32117	10.0	0.94
17	147.6	0.647	48467	15.1	0.85	42	25.3	0.771	31801	9.9	0.94
18	129.3	0.656	47163	14.7	0.86	43	24.4	0.774	31501	9.8	0.94
19	113.9	0.666	45935	14.4	0.87	44	23.6	0.776	31204	9.8	0.94
20	101.4	0.674	44792	14.0	0.87	45	22.8	0.778	30920	9.7	0.94
21	90.9	0.682	43711	13.7	0.88	46	22.1	0.780	30650	9.6	0.94
22	82.2	0.689	42724	13.4	0.89	47	21.4	0.782	30387	9.5	0.94
23	74.7	0.696	41783	13.1	0.89	48	20.8	0.785	30130	9.4	0.94
24	68.3	0.702	40912	12.8	0.89	49	20.2	0.787	29883	9.3	0.94
25	62.8	0.708	40111	12.5	0.90	50	19.6	0.788	29643	9.3	0.94

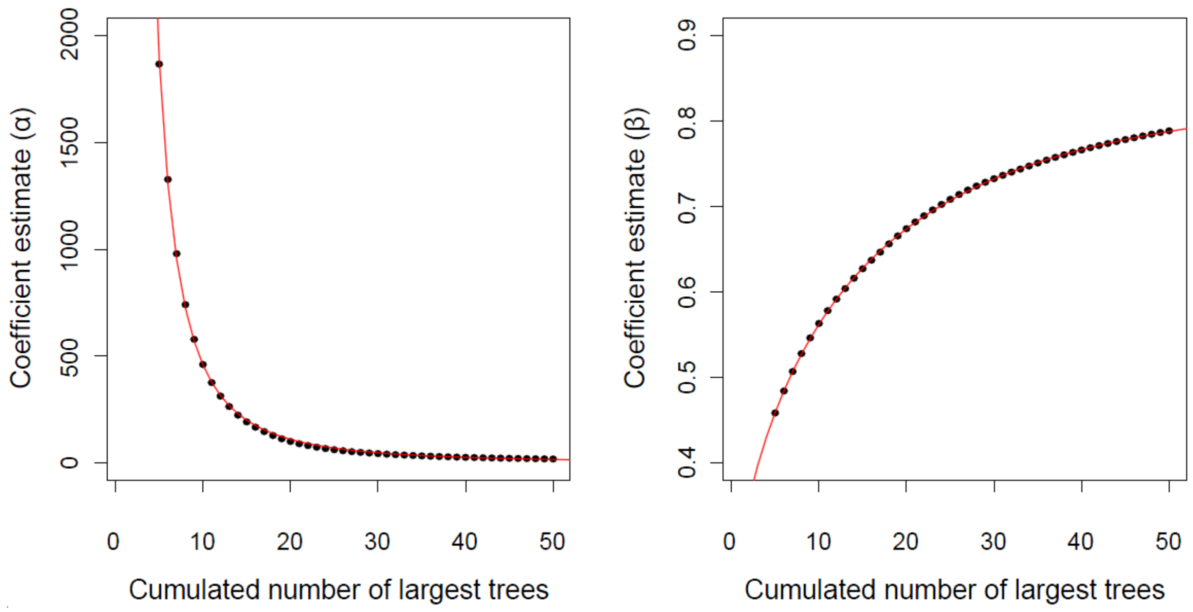

Supplementary Figure S1| Above-ground biomass (AGBtot; in kg) prediction model parameters (coefficient α and exponent β) along the cumulated number of largest trees (from the 5 largest to the 50 largest trees). The value of each coefficient is calculated based on the entire dataset of 175 1-ha plot. The coefficient α is fitted with a power model with no intercept and the exponent β is fitted with an asymptotic Weibull model (See Methods). To avoid any complex mathematical form to estimate the prediction of model parameters from any number of largest trees, the fit start with the 5 largest trees and ends with the 100 largest trees. The value of model parameters for the 1st, 2, 3 and 4 largest trees are provided in an additional table (See Supplementary Table S3).

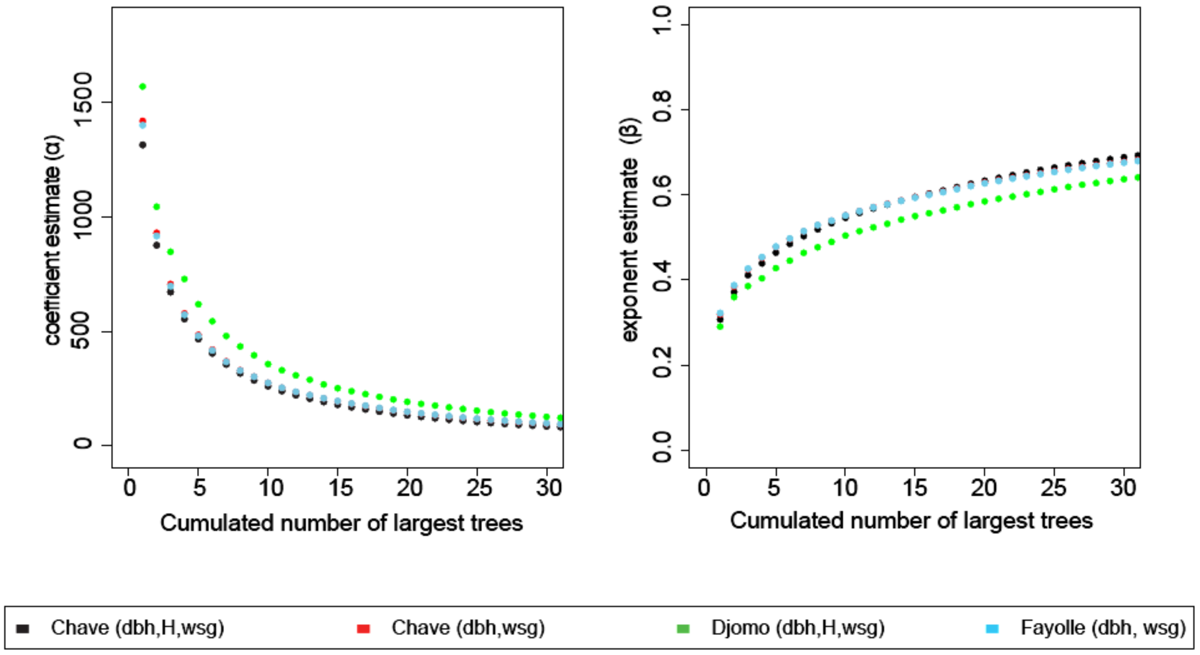

Supplementary Figure S2| Above-ground biomass (AGBtot) prediction model parameters (coefficient α and exponent β) variation along the cumulated number of largest trees considered using 4 different allometric models. The allometric models used are: Chave's model using tree height or local dbh-height relationship (black dots), Chave's model using regional dbh-height relationship (red dots), Djomo's model and Fayolle's model.

Supplementary Fig. S2 is realized using a subset of sites presenting (i) full access to the original dataset and (ii) dbh (diameter at 130cm), H (height) and WSG (wood specific gravity) measurement of each tree recorded (i.e. Mabounie, SE Cameroon, Malebo and Yangambi). A non-exhaustive list of African or pan-tropical models were used, accounting Chave's model with and without height[1] (respectively in black dots and red dots), Djomo's model from South-West Cameroon[2] (green dots) and Fayolle's model from South-East Cameroon[3]. Except from a small difference observed from Djomo's model, the figure shows there is no impact from the allometric model selected on the AGBtot prediction model we developed from largest trees.

Supplementary Table S4| Aboveground biomass regional dominant species ranked up to 80 % of cumulated AGB.

Rank	Species	Family	AGB %	Cumulated % of AGB
1	*Gilbertiodendron dewevrei*	*Fabaceae*	20.02	20.02
2	*Klainedoxa gabonensis*	*Irvingiaceae*	3.60	23.62
3	*Coula edulis*	*Olacaceae*	2.74	26.36
4	*Desbordesia glaucescens*	*Irvingiaceae*	2.72	29.08
5	*Dialium pachyphyllum*	*Fabaceae*	2.32	31.40
6	*Lecomtedoxa klaineana*	*Sapotaceae*	1.98	33.38
7	*Oubanguia alata*	*Lecythidaceae*	1.81	35.19
8	*Strombosia pustulata*	*Olacaceae*	1.81	37.00
9	*Lophira alata*	*Ochnaceae*	1.79	38.80
10	*Petersianthus macrocarpus*	*Lecythidaceae*	1.78	40.58
11	*Polyalthia cf.suaveolens*	*Annonaceae*	1.76	42.34
12	*Scorodophloeus zenkeri*	*Fabaceae*	1.74	44.08
13	*Julbernardia seretii*	*Fabaceae*	1.43	45.51
14	*Alstonia boonei*	*Apocynaceae*	1.24	46.75
15	*Pentaclethra macrophylla*	*Fabaceae*	1.21	47.95
16	*Erythrophleum suaveolens*	*Fabaceae*	1.02	48.97
17	*Staudtia kamerunensis*	*Myristicaceae*	0.99	49.96
18	*Plagiostyles africana*	*Euphorbiaceae*	0.99	50.95
19	*Strombosiopsis tetrandra*	*Olacaceae*	0.99	51.94
20	*Millettia laurentii*	*Fabaceae*	0.92	52.86
21	*Terminalia superba*	*Combretaceae*	0.87	53.73
22	*Irvingia gabonensis*	*Irvingiaceae*	0.85	54.58
23	*Mansonia altissima*	*Malvaceae*	0.76	55.33
24	*Ongokea gore*	*Olacaceae*	0.72	56.06
25	*Diogoa zenkeri*	*Olacaceae*	0.70	56.76
26	*Musanga cecropioides*	*Urticaceae*	0.70	57.46
27	*Baphia laurifolia*	*Fabaceae*	0.69	58.15
28	*Santiria trimera*	*Burseraceae*	0.68	58.84
29	*Pentaclethra eetveldeana*	*Fabaceae*	0.65	59.49
30	*Duboscia macrocarpa*	*Malvaceae*	0.65	60.14
31	*Pycnanthus angolensis*	*Myristicaceae*	0.61	60.75
32	*Chrysophyllum lacourtianum*	*Sapotaceae*	0.58	61.33
33	*Scytopetalum klaineanum*	*Lecythidaceae*	0.56	61.89
34	*Entandrophragma cylindricum*	*Meliaceae*	0.53	62.42
35	*Erythrophleum ivorense*	*Fabaceae*	0.53	62.96

36	*Protomegabaria stapfiana*	*Phyllanthaceae*	0.52	63.47
37	*Cynometra alexandri*	*Fabaceae*	0.51	63.99
38	*Drypetes*	*Putranjivaceae*	0.50	64.49
39	*Panda oleosa*	*Pandaceae*	0.48	64.97
40	*Gilletiodendron*	*Fabaceae*	0.45	65.41
41	*Pterocarpus soyauxii*	*Fabaceae*	0.43	65.84
42	*Cola lateritia*	*Malvaceae*	0.42	66.26
43	*Anonidium mannii*	*Annonaceae*	0.41	66.67
44	*Irvingia grandifolia*	*Irvingiaceae*	0.39	67.06
45	*Guarea thompsonii*	*Meliaceae*	0.38	67.44
46	*Dichostemma glaucescens*	*Euphorbiaceae*	0.37	67.81
47	*Piptadeniastrum africanum*	*Fabaceae*	0.36	68.17
48	*Pausinystalia macroceras*	*Rubiaceae*	0.36	68.53
49	*Zanthoxylum gilletii*	*Rutaceae*	0.35	68.88
50	*Baillonella toxisperma*	*Sapotaceae*	0.34	69.22
51	*Celtis zenkeri*	*Cannabaceae*	0.34	69.56
52	*Dacryodes buettneri*	*Burseraceae*	0.32	69.89
53	*Tessmannia africana*	*Fabaceae*	0.32	70.21
54	*Sacoglottis gabonensis*	*Humiriaceae*	0.32	70.53
55	*Albizia adianthifolia*	*Fabaceae*	0.31	70.84
56	*Uapaca*	*Phyllanthaceae*	0.31	71.15
57	*Garcinia punctata*	*Clusiaceae*	0.29	71.44
58	*Calpocalyx heitzii*	*Fabaceae*	0.29	71.73
59	*Copaifera*	*Fabaceae*	0.29	72.02
60	*Maranthes glabra*	*Chrysobalanaceae*	0.28	72.29
61	*Vitex*	*Lamiaceae*	0.28	72.57
62	*Hymenostegia afzelii*	*Fabaceae*	0.27	72.84
63	*Xylopia aethiopica*	*Annonaceae*	0.26	73.10
64	*Annickia chlorantha*	*Annonaceae*	0.26	73.37
65	*Bikinia evrardii*	*Fabaceae*	0.26	73.63
66	*Irvingia robur*	*Irvingiaceae*	0.26	73.89
67	*Dacryodes igaganga*	*Burseraceae*	0.26	74.14
68	*Diospyros*	*Ebenaceae*	0.26	74.40
69	*Odyendyea gabonensis*	*Simaroubaceae*	0.25	74.66
70	*Hylodendron gabunense*	*Fabaceae*	0.25	74.91
71	*Margaritaria discoidea*	*Phyllanthaceae*	0.25	75.16
72	*Trilepisium madagascariense*	*Moraceae*	0.25	75.41
73	*Symphonia globulifera*	*Clusiaceae*	0.25	75.65
74	*Albizia glaberrima*	*Fabaceae*	0.24	75.90
75	*Nauclea diderrichii*	*Rubiaceae*	0.24	76.14

76	*Triplochiton scleroxylon*	*Malvaceae*	0.24	76.37
77	*Blighia welwitschii*	*Sapindaceae*	0.23	76.61
78	*Milicia excelsa*	*Moraceae*	0.23	76.83
79	*Poga oleosa*	*Anisophylleaceae*	0.23	77.06
80	*Diospyros gabunensis*	*Ebenaceae*	0.22	77.28
81	*Cylicodiscus gabunensis*	*Fabaceae*	0.22	77.50
82	*Entandrophragma angolense*	*Meliaceae*	0.22	77.73
83	*Tridesmostemon claessensii*	*Sapotaceae*	0.22	77.94
84	*Brachystegia laurentii*	*Fabaceae*	0.22	78.16
85	*Scottellia klaineana*	*Achariaceae*	0.22	78.38
86	*Dialium guineense*	*Fabaceae*	0.21	78.59
87	*Strephonema pseudocola*	*Combretaceae*	0.21	78.80
88	*Cynometra hankei*	*Fabaceae*	0.21	79.01
89	*Anthonotha macrophylla*	*Fabaceae*	0.21	79.22
90	*Dialium pentandrum*	*Fabaceae*	0.21	79.42
91	*Erythrophleum lasianthum*	*Fabaceae*	0.21	79.63
92	*Celtis philippensis*	*Cannabaceae*	0.21	79.84
93	*Tabernaemontana crassa*	*Apocynaceae*	0.21	80.04

Supplementary Table S5| Aboveground biomass local hyperdominant species.

Site	Rank	Species	Family	AGB %	Cumulated % of AGB
Korup	1	*Lecomtedoxa klaineana*	*Sapotaceae*	13.92	13.92
	2	*Oubanguia alata*	*Lecythidaceae*	12.74	26.66
	3	*Desbordesia glaucescens*	*Irvingiaceae*	5.27	31.93
	4	*Protomegabaria stapfiana*	*Phyllanthaceae*	3.63	35.57
	5	*Erythrophleum ivorense*	*Fabaceae*	3.55	39.11
	6	*Strombosia pustulata*	*Olacaceae*	2.51	41.62
	7	*Dichostemma glaucescens*	*Euphorbiaceae*	2.28	43.90
	8	*Hymenostegia afzelii*	*Fabaceae*	1.87	45.77
	9	*Vitex spp.*	*Lamiaceae*	1.83	47.60
	10	*Staudtia kamerunensis*	*Myristicaceae*	1.71	49.31
	11	*Lophira alata*	*Ochnaceae*	1.58	50.89
Ngovayang	1	*Tetraberlinia bifoliolata*	*Fabaceae*	7.37	7.37
	2	*Coula edulis*	*Coulaceae*	4.06	11.43
	3	*Guibourtia tessmannii*	*Fabaceae*	3.58	15.01
	4	*Coelocaryon preussii*	*Myristicaceae*	3.18	18.19

	5	*Desbordesia glaucescens*	*Irvingiaceae*	3.10	21.29
	6	*Allanblackia floribunda*	*Clusiaceae*	2.63	23.92
	7	*Staudtia kamerunensis*	*Myristicaceae*	2.53	26.45
	8	*Brachystegia cynometroides*	*Fabaceae*	2.46	28.91
	9	*Plagiosiphon emarginatus*	*Fabaceae*	2.29	31.20
	10	*Plagiostyles africana*	*Euphorbiaceae*	2.16	33.35
	11	*Pycnanthus angolensis*	*Myristicaceae*	1.94	35.29
	12	*Scyphocephalium mannii*	*Myristicaceae*	1.90	37.18
	13	*Santiria trimera*	*Burseraceae*	1.86	39.04
	14	*Piptadeniastrum africanum*	*Fabaceae*	1.82	40.87
	15	*Pentadesma grandifolia*	*Clusiaceae*	1.33	42.19
	16	*Dacryodes klaineana*	*Burseraceae*	1.23	43.43
	17	*Dialium pachyphyllum*	*Fabaceae*	1.10	44.53
	18	*Baillonella toxisperma*	*Sapotaceae*	1.04	45.57
	19	*Treculia obovoidea*	*Moraceae*	1.00	46.56
	20	*Poga oleosa*	*Anisophylleaceae*	0.94	47.50
	21	*Strombosia scheffleri*	*Strombosiaceae*	0.93	48.44
	22	*Dialium dinklagei*	*Fabaceae*	0.92	49.36
	23	*Strombosiopsis tetrandra*	*Strombosiaceae*	0.88	50.24
Mabounie	1	*Coula edulis*	*Olacaceae*	14.66	14.66
	2	*Lophira alata*	*Ochnaceae*	8.40	23.06
	3	*Dialium pachyphyllum*	*Fabaceae*	8.38	31.44
	4	*Desbordesia glaucescens*	*Irvingiaceae*	7.17	38.62
	5	*Diogoa zenkeri*	*Olacaceae*	3.37	41.99
	6	*Santiria trimera*	*Burseraceae*	2.58	44.57
	7	*Scytopetalum klaineanum*	*Lecythidaceae*	2.52	47.09
	8	*Gilletiodendron spp.*	*Fabaceae*	2.30	49.39
	9	*Dacryodes buettneri*	*Burseraceae*	1.74	51.13
SE Cameroon	1	*Petersianthus macrocarpus*	*Lecythidaceae*	5.63	5.63
	2	*Terminalia superba*	*Combretaceae*	4.63	10.26
	3	*Pentaclethra macrophylla*	*Fabaceae*	4.45	14.71
	4	*Alstonia boonei*	*Apocynaceae*	4.31	19.02
	5	*Mansonia altissima*	*Malvaceae*	4.03	23.05
	6	*Erythrophleum suaveolens*	*Fabaceae*	3.70	26.75
	7	*Polyalthia cf.suaveolens*	*Annonaceae*	3.68	30.44

	8	*Baphia laurifolia*	*Fabaceae*	3.58	34.01
	9	*Klainedoxa gabonensis*	*Irvingiaceae*	3.45	37.46
	10	*Desbordesia glaucescens*	*Irvingiaceae*	3.34	40.80
	11	*Entandrophragma cylindricum*	*Meliaceae*	2.54	43.35
	12	*Duboscia macrocarpa*	*Malvaceae*	2.35	45.69
	13	*Celtis zenkeri*	*Cannabaceae*	1.78	47.48
	14	*Strombosia pustulata*	*Olacaceae*	1.69	49.17
	15	*Irvingia gabonensis*	*Irvingiaceae*	1.52	50.68
Mbaïki	1	*Entandrophragma cylindricum*	*Meliaceae*	6.66	6.66
	2	*Celtis tessmannii*	*Cannabaceae*	6.27	12.93
	3	*Celtis zenkeri*	*Cannabaceae*	5.85	18.78
	4	*Petersianthus macrocarpus*	*Lecythidaceae*	5.38	24.16
	5	*Terminalia superba*	*Combretaceae*	3.84	28.00
	6	*Triplochiton scleroxylon*	*Malvaceae*	3.72	31.73
	7	*Trilepisium madagascariense*	*Moraceae*	3.18	34.91
	8	*Staudtia kamerunensis*	*Myristicaceae*	2.23	37.14
	9	*Albizia zygia*	*Fabaceae*	1.90	39.04
	10	*Manilkara mabokeensis*	*Sapotaceae*	1.62	40.66
	11	*Manilkara fouilloyana*	*Sapotaceae*	1.47	42.13
	12	*Klainedoxa gabonensis*	*Irvingiaceae*	1.46	43.59
	13	*Entandrophragma angolense*	*Meliaceae*	1.36	44.95
	14	*Irvingia grandifolia*	*Irvingiaceae*	1.34	46.29
	15	*Strombosiopsis tetrandra*	*Olacaceae*	1.28	47.57
	16	*Musanga cecropioides*	*Moraceae*	1.27	48.84
	17	*Manilkara aubrevillei*	*Sapotaceae*	1.21	50.06
Malebo	1	*Klainedoxa gabonensis*	*Irvingiaceae*	16.17	16.17
	2	*Millettia laurentii*	*Fabaceae*	6.10	22.27
	3	*Strombosia pustulata*	*Olacaceae*	6.02	28.29
	4	*Plagiostyles africana*	*Euphorbiaceae*	5.58	33.87
	5	*Pentaclethra eetveldeana*	*Fabaceae*	4.42	38.29
	6	*Polyalthia cf.suaveolens*	*Annonaceae*	3.91	42.20
	7	*Gilbertiodendron dewevrei*	*Fabaceae*	3.70	45.90
	8	*Dialium pachyphyllum*	*Fabaceae*	3.68	49.57
	9	*Scorodophloeus zenkeri*	*Fabaceae*	2.20	51.77

Yangambi	1	*Gilbertiodendron dewevrei*	*Fabaceae*	27.33	27.33
	2	*Scorodophloeus zenkeri*	*Fabaceae*	9.36	36.69
	3	*Petersianthus macrocarpus*	*Lecythidaceae*	4.87	41.56
	4	*Musanga cecropioides*	*Urticaceae*	2.65	44.21
	5	*Panda oleosa*	*Pandaceae*	1.94	46.15
	6	*Trilepisium madagascariense*	*Moraceae*	1.83	47.98
	7	*Tridesmostemon claessensii*	*Sapotaceae*	1.65	49.62
	8	*Strombosiopsis tetrandra*	*Olacaceae*	1.58	51.20
Ituri-lenda	1	*Gilbertiodendron dewevrei*	*Fabaceae*	76.71	76.71

3) Supplementary Note

In Practice

The fact that only 5 % of the stems can predict 87 % of the variation in AGB_{TOT} across the study sites in Central Africa has direct implications for assessments of forest carbon stocks and monitoring as required to reduce the emission for deforestation and forest degradation[4] (REDD+). From an operational point of view, the 20 largest trees present DBHs greater than or equal to 50 cm (DBH ≥ 50 cm) in more than 90 % of our plots. Therefore, one can measure and identify all of the stems with a DBH ≥ 50 cm and ensure a prediction of AGB with a minimum R^2 of 0.87 and a maximum relative RSE of 14 % in natural forests. This result indicates that the inventories from forest logging companies may be sufficient to accurately assess their forest AGB using a standardised approach. Yet, we recommend that particular model should be implemented in forests being recently or currently logged, as we expect a substantial shift in the relationship between the biomass of the largest trees and the total biomass after removal of the trees of interest.

Consequently, we suggest the development of a cost-effective field sampling strategy that decreases the effort of field campaigns by focusing on the largest trees while covering a larger sampling area to minimise spatial sampling errors. Such a design could also improve the calibration of remote-sensing products by solving their main reported problem: the lack of an extensive but dense ground-truthed sampling design[5]. In addition, our results suggest that remote-sensing techniques should focus on canopy trees and provide support for methods that rely on forest height canopy profiles[6,7] or forest canopy horizontal spatial heterogeneity[8,9] to predict forest AGB. Our results may also help define priorities for the construction of improved regional allometric equations for tree-level estimations of AGB by focusing efforts on the peculiarities of large trees and AGB hyperdominant species.

References

1. Chave, J. *et al.* Tree allometry and improved estimation of carbon stocks and balance in tropical forests. *Oecologia* **145,** 87–99 (2005).

2. Djomo, A. N., Ibrahima, A., Saborowski, J. & Gravenhorst, G. Allometric equations for biomass estimations in Cameroon and pan moist tropical equations including biomass data from Africa. *For. Ecol. Manage.* **260,** 1873–1885 (2010).

3. Fayolle, A., Doucet, J.-L., Gillet, J.-F., Bourland, N. & Lejeune, P. Tree allometry in Central Africa: Testing the validity of pantropical multi-species allometric equations for estimating biomass and carbon stocks. *For. Ecol. Manage.* **305,** 29–37 (2013).

4. Agrawal, A., Nepstad, D. & Chhatre, A. Reducing Emissions from Deforestation and Forest Degradation. *Annu. Rev. Environ. Resour.* **36,** 373–396 (2011).

5. Mitchard, E. T. A., Saatchi, S. S., Asner, G. P. & Baccini, A. Uncertainty in the spatial distribution of tropical forest biomass: a comparison of pan-tropical maps. *Carbon Balance Manag.* **8,** 1–13 (2013).

6. Zolkos, S. G., Goetz, S. J. & Dubayah, R. A meta-analysis of terrestrial aboveground biomass estimation using lidar remote sensing. *Remote Sens. Environ.* **128,** 289–298 (2013).

7. Mascaro, J., Detto, M., Asner, G. P. & Muller-Landau, H. C. Evaluating uncertainty in mapping forest carbon with airborne LiDAR. *Remote Sens. Environ.* **115,** 3770–3774 (2011).

8. Barbier, N., Couteron, P., Gastelly-Etchegorry, J.-P. & Proisy, C. Linking canopy images to forest structural parameters: potential of a modeling framework. *Ann. For. Sci.* **69,** 305–311 (2012).

9. Bastin, J.-F. *et al.* Aboveground biomass mapping of African forest mosaics using canopy texture analysis: toward a regional approach. *Ecol. Appl.* **24,** 1984–2001 (2014).
